# 

**Published:** 1850-09

**Authors:** 


### WESTERN JOURNAL

### OF

### MEDICINE AND SURGERY:

EDITED BY

LUNSFORD P. YANDELL, M. D.,

PROFESSOR OF PHYSIOLOGY AND PATHOLOGICAL ANATOMY IN THE UNIVERSITY OF LOUISVILLE,

AND

THEODORE S. BELL, M. D.

---

CXXIX.—SEPTEMBER, 1850.

---

**THIRD SERIES.**

Vol. VI.....No. III.

---

LOUISVILLE, KY:

PUBLISHED MONTHLY BY PRENTICE & WEISSINGER,

PROPRIETORS AND PRINTERS.

1850.

[POSTAGE 6¼ CENTS.]

